# Supplementary material for: The RNA helicase UPF1 associates with mRNAs co-transcriptionally and is required for the release of mRNAs from gene loci
Source: eLife. 2019 Mar 25;8:e41444. doi: 10.7554/eLife.41444 (PMC6447362; doi:10.7554/eLife.41444)
Supplement: Supplementary file 1. [file elife-41444-supp1.doc]

**Supplementary file 1**. Peptides used for UPF1 antibody generation (positions refer to full length sequence FBpp0073433)

| **No.** | **Start** | **End** | **Peptide** |
| --- | --- | --- | --- |
| 1 | 43 | 52 | TSQSQTQNDQ |
| 2 | 85 | 94 | DEPGSSYVKE |
| 3 | 1084 | 1093 | PGGNKKTNKL |
| 4 | 342 | 351 | HYVGELYNPW |
| 5 | 788 | 797 | QYQGSLHSRL |
| 6 | 27 | 36 | DTQPTQYDYR |
| 7 | 528 | 537 | KSREAIDSPV |
| 8 | 60 | 69 | SAGDSHPRLA |
| 9 | 1140 | 1149 | SQQPELSQDF |
| 10 | 259 | 268 | KPGIDSEPAH |
| 11 | 1060 | 1069 | QTGNFSPGNS |
| 12 | 1117 | 1126 | AAPYSQHPMP |
