# Supplementary material for: The RNA helicase UPF1 associates with mRNAs co-transcriptionally and is required for the release of mRNAs from gene loci
Source: eLife. 2019 Mar 25;8:e41444. doi: 10.7554/eLife.41444 (PMC6447362; doi:10.7554/eLife.41444)
Supplement: Supplementary file 2. [file elife-41444-supp2.doc]

**Supplementary file 2.** Most enriched transcription units by UPF1 ChIP-seq (black=Protein coding genes, red=Noncoding RNAs).

| **No.** | **Transcript** | **Chromosome** | **Cytological Location** | **Strand** | **Gene** | **UPF1 Enrichment** |
| --- | --- | --- | --- | --- | --- | --- |
| 1 | NR_002554 | chr2R | 60A3 | - | snoRNA:Psi18S-176 | 20.92784248 |
| 2 | NR_073860 | chr2R | 45F1 | + | CR43651 | 19.84962099 |
| 3 | NR_073861 | chr2R | 45F1 | + | CR43651 | 19.58870612 |
| 4 | NM_078901 | chr2R | 42A7 | - | Act42A | 16.53573972 |
| 5 | NM_166890 | chrX | 2B1 | - | sta | 15.7976555 |
| 6 | NM_001297856 | chrX | 2B1 | - | sta | 14.65188523 |
| 7 | NM_057402 | chrX | 2B1 | - | sta | 14.65188523 |
| 8 | NM_001316458 | chr3L | 70A6 | + | Nplp2 | 14.2141182 |
| 9 | NM_144173 | chr3L | 70A6 | + | Nplp2 | 14.2141182 |
| 10 | NM_166889 | chrX | 2B1 | - | sta | 14.0181416 |
| 11 | NM_206272 | chr3L | 63F5 | - | Ubi-p63E | 13.10522634 |
| 12 | NR_001992 | chr3R | 84A5 | - | 7SLRNA:CR32864 | 13.04190544 |
| 13 | NR_037753 | chr3R | 84A5 | - | 7SLRNA:CR42652 | 13.04190544 |
| 14 | NM_001274454 | chr3L | 63F5 | - | Ubi-p63E | 12.94301365 |
| 15 | NM_168043 | chr3L | 63F5 | - | Ubi-p63E | 12.86980148 |
| 16 | NM_079185 | chr3L | 63F5 | - | Ubi-p63E | 12.8624403 |
| 17 | NM_001299392 | chr2R | 48C5 | + | Ef1alpha48D | 12.7336405 |
| 18 | NR_002067 | chr3R | 93D | + | Hsromega | 12.6383098 |
| 19 | NR_002069 | chr3R | 93D | + | Hsromega | 12.6383098 |
| 20 | NM_001300130 | chr3L | 70A6 | + | Nplp2 | 12.582198 |
| 21 | NM_001316502 | chr3R | 86D8 | + | Tctp | 11.88147055 |
| 22 | NM_058027 | chr2R | 48C5 | + | Ef1alpha48D | 11.51812571 |
| 23 | NM_001014455 | chr2L | 21 E2 | + | PNUTS | 11.50911291 |
| 24 | NR_048444 | chr3R | 93D | + | Hsromega | 11.47018513 |
| 25 | NM_001297855 | chrX | 2B1 | + | rush | 11.45428524 |
| 26 | NM_130567 | chrX | 2B1 | + | rush | 11.36293998 |
| 27 | NM_170070 | chr3R | 94 E10-94 13 | - | pnt | 11.36070171 |
| 28 | NM_141791 | chr3R | 86D8 | + | Tctp | 11.3160895 |
| 29 | NM_001298659 | chr2L | 23F3-23F6 | + | Thor | 11.29086613 |
| 30 | NM_057284 | chr3R | 93 E13 | + | RpS3 | 10.9579823 |
| 31 | NM_001014454 | chr2L | 21 E2 | + | PNUTS | 10.86549487 |
| 32 | NM_001298210 | chrX | 10-E3 to 10 E4 | - | Hsc70-3 | 10.85467784 |
| 33 | NR_133429 | chr3R | 93D | + | Hsromega | 10.74841913 |
| 34 | NM_167307 | chrX | 10-E3 to 10 E4 | - | Hsc70-3 | 10.70276539 |
| 35 | NM_165850 | chr2R | 48C5 | + | Ef1alpha48D | 10.69805745 |
| 36 | NM_001202085 | chr3L | 61B2 | - | CG42846 | 10.5511492 |
| 37 | NM_001275943 | chr3R | 94 E10-94 13 | - | pnt | 10.42349043 |
| 38 | NM_079983 | chr2R | 57C3 | + | Xbp1 | 10.40232605 |
| 39 | NM_166427 | chr2R | 57C3 | + | Xbp1 | 10.39685831 |
| 40 | NM_001202063 | chr2R | 59B6 | - | CG3800 | 10.39235699 |
| 41 | NM_079175 | chr3L | 63B11 | + | Hsp83 | 10.35797394 |
| 42 | NM_001299393 | chr2R | 48C5 | + | Ef1alpha48D | 10.3424767 |
| 43 | NM_143714 | chrX | 5D2 | - | mab-21 | 10.22106132 |
| 44 | NM_167180 | chrX | 8C14 | - | His3.3B | 10.19996373 |
| 45 | NM_057947 | chr2L | 23F3-23F6 | + | Thor | 10.10779488 |
| 46 | NM_001103474 | chrX | 9D2 | - | spri | 10.09939045 |
| 47 | NM_001272490 | chrX | 9D2 | - | spri | 10.0968423 |
| 48 | NM_001272491 | chrX | 9D2 | - | spri | 10.0968423 |
| 49 | NM_001298516 | chrX | 18D3 | + | RpS10b | 10.05584386 |
| 50 | NM_001274433 | chr3L | 63B11 | + | Hsp83 | 9.962334214 |
| 51 | NM_167065 | chrX | 5 E4 | - | Ubi-p5E | 9.845904641 |
| 52 | NM_167306 | chrX | 10 E3-10 E4 | - | Hsc70-3 | 9.739493741 |
| 53 | NM_167308 | chrX | 10 E3-10 E4 | - | Hsc70-3 | 9.739493741 |
| 54 | NM_206223 | chr3L | 61B2 | + | CG33229 | 9.726778964 |
| 55 | NM_169625 | chr3R | 88 E4 | + | Hsc70-4 | 9.714744345 |
| 56 | NM_001300552 | chr3R | 94 E13 | + | RpS3 | 9.705903021 |
| 57 | NM_176503 | chr3R | 88 E4 | + | Hsc70-4 | 9.687864086 |
| 58 | NM_001347750 | chrX | 2B1 | + | mei-38 | 9.661728876 |
| 59 | NM_078497 | chrX | 5C7 | + | Act5C | 9.593373151 |
| 60 | NM_168070 | chr3L | 64A10 | - | ImpL2 | 9.571757664 |
| 61 | NM_134543 | chrX | 19C5 | + | l(1)G0004 | 9.506154816 |
| 62 | NM_169841 | chr3R | 91D3 | + | Xrp1 | 9.489137734 |
| 63 | NM_079879 | chr4 | 101 F1 | - | RpS3A | 9.472988193 |
| 64 | NM_166714 | chr4 | 101 F1 | - | RpS3A | 9.472988193 |
| 65 | NM_001275657 | chr3R | 88 E4 | + | Hsc70-4 | 9.464906214 |
| 66 | NM_001298008 | chrX | 5 E4 | - | Ubi-p5E | 9.44989544 |
| 67 | NM_169626 | chr3R | 88 E4 | + | Hsc70-4 | 9.391845758 |
| 68 | NM_176502 | chr3R | 88 E4 | + | Hsc70-4 | 9.391845758 |
| 69 | NM_176719 | chrX | 8C14 | - | His3.3B | 9.373510295 |
| 70 | NM_169627 | chr3R | 88 E4 | + | Hsc70-4 | 9.333994534 |
| 71 | NM_137895 | chr2R | 59B6 | - | CG3800 | 9.317056284 |
| 72 | NM_001272174 | chrX | 1C5 | + | Sec22 | 9.263596844 |
| 73 | NM_130507 | chrX | 1C5 | + | Sec22 | 9.263596844 |
| 74 | NM_164617 | chr2L | 25C1 | + | Col4a1 | 9.208538686 |
| 75 | NM_057786 | chrX | 1C4 | - | RpL22 | 9.203239849 |
| 76 | NM_164615 | chr2L | 25C1 | + | Col4a1 | 9.190852727 |
| 77 | NM_165869 | chr2R | 48 E8-48 E9 | - | RpS11 | 9.137225506 |
| 78 | NM_001014453 | chr2L | 21 E2 | + | PNUTS | 9.022927536 |
| 79 | NM_001272921 | chr2L | 21 E2 | + | PNUTS | 9.022927536 |
| 80 | NM_164616 | chr2L | 25C1 | + | Col4a1 | 9.013677552 |
| 81 | NM_001275501 | chr3R | 85 E1 | - | Calr | 9.013384298 |
| 82 | NM_001014725 | chrX | 5C7 | + | Act5C | 8.989781735 |
| 83 | NM_132656 | chrX | 11F1 | + | CG1673 | 8.981761268 |
| 84 | NM_079569 | chr3R | 85 E1 | - | Calr | 8.981419499 |
| 85 | NM_079632 | chr3R | 88 E4 | + | Hsc70-4 | 8.957987156 |
| 86 | NM_001273288 | chr2L | 85 E1 | - | Rack1 | 8.945814985 |
| 87 | NM_057921 | chr2L | 85 E1 | - | Rack1 | 8.945814985 |
| 88 | NM_132800 | chrX | 13C4 | - | CG15642 | 8.919897052 |
| 89 | NM_001014726 | chrX | 5C7 | + | Act5C | 8.909849299 |
| 90 | NM_001299958 | chr3L | 61B2 | + | CG33229 | 8.850750946 |
| 91 | NM_079355 | chr3L | 71B5 | - | Pdi | 8.830574597 |
| 92 | NM_080366 | chr2L | 39 E7 | - | EF2 | 8.804229445 |
| 93 | NM_001298794 | chr2L | 85 E1 | - | Rack1 | 8.793822499 |
| 94 | NM_165395 | chr2L | 39 E7 | - | EF2 | 8.793744101 |
| 95 | NM_165394 | chr2L | 39 E7 | - | EF2 | 8.732148442 |
| 96 | NR_123764 | chrX | 1C4 | + | CR44965 | 8.355359116 |
| 97 | NR_002545 | chr2R | 54A2 | + | snoRNA:U3:54Ab | 8.034844038 |
| 98 | NR_002544 | chr2R | 54A2 | + | snoRNA:U3:54Aa | 7.685229313 |
| 99 | NR_002493 | chr3R | 84D4 | - | snRNA:7SK | 7.602249655 |
| 100 | NR_073616 | chr4 | 102B7 | + | CR44027 | 7.389972024 |
| 101 | NR_133116 | chr2R | 50A1-A3 | - | CR44206 | 7.36480527 |
| 102 | NR_047918 | chr2L | 27F4 | + | mir-305 | 7.207345421 |
