# Supplementary material for: The RNA helicase UPF1 associates with mRNAs co-transcriptionally and is required for the release of mRNAs from gene loci
Source: eLife. 2019 Mar 25;8:e41444. doi: 10.7554/eLife.41444 (PMC6447362; doi:10.7554/eLife.41444)
Supplement: Supplementary file 3. [file elife-41444-supp3.doc]

**Supplementary file 3.** List of PCR primers used in present study (bold=T7 promoter region)

| **No** | **Primer Name** | **Primer Sequence** |
| --- | --- | --- |
| 1 | UPF1 RNAi (F) | 5’-**TTAATACGACTCACTATAGGGGAGA**GGAGAAGCCAGGCATTGA-3’ |
| 2 | UPF1 RNA (R) | 5’-**TTAATACGACTCACTATAGGGGAGA**GACCGTGGCCCAACAGG-3’ |
| 3 | Y14 RNAi (F) | 5’-**TTAATACGACTCACTATAGGGGAGA**CGATGTGTTGGACATTGACA-3’ |
| 4 | Y14 RNAi (R) | 5’-**TTAATACGACTCACTATAGGGGAGA**GACGCTTTTCGGACTTTTT-3’ |
| 5 | eIF4AIII RNAi (F) | 5’-**TTAATACGACTCACTATAGGGGAGA**GACGAATTGACACTGGAAGG-3’ |
| 6 | eIF4AIII RNAi (R) | 5’-**TTAATACGACTCACTATAGGGGAGA**AGAATATTAGTTTAGATCAAGTCAG-3’ |
| 7 | snRNPU1 70K RNAi (F) | 5’-**TTAATACGACTCACTATAGGGGAGA**AGCACGCCGATGGTAAG-3’ |
| 8 | snRNPU1 70K RNAi (R) | 5’-**TTAATACGACTCACTATAGGGGAGA**TGGAGCGCTTCTTTTTCTT-3’ |
| 9 | snRNPU1 70K (F) | 5’-CTACGGGCCCATCAAGAAGA-3’ |
| 10 | snRNPU1 70K (R) | 5’-CGCTTGCTGTCGATCTTCTT-3’ |
| 11 | RpL23A s (F) | 5’-GGCAATCGAATTTGTGCGAA-3’ |
| 12 | RpL23A s (R) | 5’-GATTGCTCACTCGGTTGCTA-3’ |
| 13 | RpL23A m (F) | 5’-TCGAACAGATCATCAAGGGC-3’ |
| 14 | RpL23A m (R) | 5’-CGATTTCCTGGGGTACTTGG-3’ |
| 15 | RpL23A e (F) | 5’-TAACGTGTGGAATGGGCATC-3’ |
| 16 | RpL23A e (R) | 5’-GAAACGTTCATCCTCGCTCT-3’ |
| 17 | Xrp1 E1 (F) | 5’-AGATCCTACGACGCTCCAAG-3’ |
| 18 | Xrp1 E1 (R) | 5’-TGCTCCTTATCTCCGGTGAC-3’ |
| 19 | Xrp1 E3 (F) | 5’-TCATAATGCTTGTGGGGCCT-3’ |
| 20 | Xrp1 E3 (R) | 5’-AGGGTCCCTCTAAACAAGCT-3’ |
| 21 | Xrp1 I3 (F) | 5’-AACCACGTAACCACCCATCT-3’ |
| 22 | Xrp1 I3 (R) | 5’-TATCTTGGGTTGGCTTGGGT-3’ |
| 23 | Xrp1 E4 (F) | 5’-GCAGCAACAGCAACAACAAC-3’ |
| 24 | Xrp1 E4 (R) | 5’-TTGAGATACGGCACCTCCTC-3’ |
| 25 | Xrp1 I4 (F) | 5’-TTCTAACTTGTTGCGCCGAC-3’ |
| 26 | Xrp1 I4 (R) | 5’-TCAGATTTATCCGCGGACCA-3’ |
| 27 | Xrp1 E6 (F) | 5’-CGTTGAAGAAGTCGAGAAGCA-3’ |
| 28 | Xrp1 E6 (R) | 5’-TAAACACTCCTCGCGCACTA-3’ |
| 29 | Socs36E (F) | 5’-CAGAAAACCGCACACAGACA-3’ |
| 30 | Socs36E (R) | 5’-CACACATCGGACTAACAGCG-3’ |
| 31 | RpS12 e (F) | 5’-AAGATCGACAAGGAGGGCAA-3’ |
| 32 | RpS12 e (R) | 5’-GTTCTGCCTGAGATGGTCCT-3’ |
| 33 | Intergenic 2R (F) | 5’-TCGTATCGCTGGCACTGTAA-3’ |
| 34 | Intergenic 2R (R) | 5’-ATATAGCCCGTGCCAGGAGT-3’ |
| 36 | 18s RNA (F) | 5’-ACCGGTGGAGTTCTTATATGTGAT-3’ |
| 37 | 18s RNA (R) | 5’-CGGCCCACAATAACACTCGT-3’ |
